# Herbal extracts that induce type I interferons through Toll-like receptor 4 signaling

**DOI:** 10.29219/fnr.v66.5524

**Published:** 2022-01-28

**Authors:** Misa Nakasuji-Togi, Sumihito Togi, Keita Saeki, Yasuhiko Kojima, Keiko Ozato

**Affiliations:** 1 Division of Developmental Biology, Eunice Kennedy Institute of Child Health and Human Development, National Institutes of Health, MD, USA; 2 Department of Regenerative Medicine, School of Medicine, Kanazawa Medical University, Ishikawa, Japan; 3 Center for Regenerative Medicine, Kanazawa Medical University Hospital, Ishikawa, Japan; 4 Division of Genomic Medicine, Department of Advanced Medicine, Medical Research Institute, Kanazawa Medical University, Ishikawa, Japan; 5 Center for Clinical Genomics, Kanazawa Medical University Hospital, Ishikawa, Japan; 6 NPO Interferon Herb Research Institute, Tokyo, Japan

**Keywords:** herbal medicine, interferon, pathogen resistance, macrophages, Toll-like receptors

## Abstract

**Background:**

A mixture of five herbal extracts called internatural (INT), which is prepared from pumpkin seeds, purple turmeric, pearl barley, corn pistil, and cinnamon, is widely used by people in Japan and elsewhere for its immunity-enhancing effects and general health. Although anecdotal evidence indicates its efficacy, the mechanisms by which INT boosts immunity have remained unknown.

**Objective:**

The aim of this study was to investigate whether INT induces type I interferons (IFNs) in murine bone marrow-derived macrophages (BMDMs) and by what mechanism.

**Design:**

We measured induction of type I IFNs (IFNβ and IFNα) in BMDMs treated with INT or other Toll-like receptor ligands: bacterial lipopolysaccharides (LPS), dsRNA, poly(I:C), and CpG oligonucleotides. To investigate whether INT signals through Toll-like receptor 4 (TLR4), we tested TLR4-specific inhibitor. We also tested if INT utilizes TLR4 adaptors, toll/IL-1 receptor (TIR) domain-containing adaptor (TRIF), or myeloid differentiation factor 88 (MyD88), we examined INT induction of IFNβ in TRIF-KO and MyD88-KO BMDMs. We then investigated whether INT provides an antiviral effect upon fibroblasts either directly or indirectly using the encephalomyocarditis virus (EMCV) model.

**Results:**

We first observed that INT, when added to BMDMs, potently induces type I IFNs (IFNβ and IFNα) within 2 h. INT induction of IFN expression was mediated by TLR4, which signaled through the TRIF/MyD88 adaptors, similar to LPS. A high-molecular-weight fraction (MW > 10,000) of INT extracts contained IFN-inducing activity. Supernatants from INT-treated BMDMs protected untreated fibroblast from EMCV infection as reduced viral titers.

**Conclusions:**

INT induced type I IFN mRNA and proteins in BMDMs and other cell types. This induction was mediated by TLR4, which transduces signals using the TRIF/MyD88 pathway. The high-MW component of INT contained type I IFN inducing activity. The supernatants from INT-treated cells displayed antiviral activity and protected cells from EMCV infection. These findings indicate that INT is a novel natural IFN inducer that strengthens host’s innate immunity.

## Popular scientific summary

- Internatural (INT) is a widely used herbal medicine composed of extracts from five select plants.
- INT potently induced type I interferons (IFNs) in macrophages, dendritic cells, and fibroblasts.
- INT induced type I IFN gene expression through the Toll-like receptor 4 and TRIF/MyD88 pathway.
- A high-molecular-weight fraction (MW > 10,000) of INT contained type I IFN-inducing activity.
- INT conferred protection against viral infection upon fibroblasts.

Type I interferons (IFNs) are cytokines that elicit broad anti-pathogen activities (1, 2). There are more than 10 type I IFN genes that are clustered in chromosomes 9 and 4 in humans and mice, respectively (3, 4). These genes are activated in response to viruses, pathogens, and their breakdown components, which engage pathogen recognition receptors such as Toll-like receptors (TLRs) (5–8). These ligand–receptor interactions first activate the expression of IFNβ, followed by induction of IFNα genes (9, 10). Type I IFNs afford innate resistance against RNA viruses, DNA viruses, retroviruses, a wide range of bacteria, and pathogenic fungi (2). Type I IFNs enhance innate and adaptive immunity and contribute to the control of cancer cell growth (11, 12).

Type I IFN genes are activated by natural products (13–19) such as herbal products that have long been used to treat diverse human aliments, a practice that predates the written human history (13, 14). Extracts from a wide array of plants, including aloe and kelp, are reported to exhibit antiviral and IFN-inducing activity (15–17) or to enhance the potencies of IFN (18). Other herbal products possess antitumor activities (19).

Internatural (INT) was developed in the 1950s by Yasuhiko Kojima, who reported an antiviral activity in rabbits infected with vaccinia virus (20, 21), prior to the discovery of type I IFN by Issacs and Lindenmann (22). Kojima’s subsequent efforts led to the discovery and isolation of herbal mixtures with IFN-inducing activity. The final product, INT, comprises extracts prepared from pumpkin seeds, purple turmeric, pearl barley, corn pistil, and cinnamon. These plant substances were selected after screening hundreds of other plants. Today, INT, which is manufactured by Paladium Corp. (http://paladium.co.jp/about/ifninducer/#feature) under strict quality control, is distributed in Japan and other countries in the world as an oral supplement to enhance general immunity.

Although the antiviral activity of INT was indicated in the rabbit vaccinia model, INT’s biological activity and underlying mechanisms of action have remained elusive. Here, we report that INT induces IFNβ and IFNα gene expressions in murine bone marrow-derived macrophages (BMDMs) as well as in other cells and confers protection against viral infection. We show that INT interacts with and signals through TLR4 similar to bacterial lipopolysaccharide (LPS). These findings provide compelling evidence that INT is a powerful inducer of IFN to strengthen the host’s innate immunity.

## Materials and methods

### Materials

A lyophilized powder of INT (1.0 g) (Paladium Corp., Japan) was reconstituted in 10 mL of distilled water, heated at 90°C for 5 min, and centrifuged at 3,000 rpm for 10 min. The undiluted extract was applied to an Amicon Ultra-2 Centrifugal Filter 10 K device (Millipore, Billerica, MA, United States) and processed according to the manufacturer’s instructions. LPS (from *Escherichia coli* 0111: B4), CpG (1826 ODN and 1585 ODN), polyinosine-polycytidylic acid [poly(I:C)] high-molecular-weight (HMW), and the transfection reagent LyoVec™ were purchased from InvivoGen (San Diego, CA, United States). These reagents were added to BMDMs and other cells at the concentrations as follows: INT (500-, 1,000-, or 5,000-fold dilution), LPS (200 ng/mL), poly(I:C) (1 μg/mL), and CpG ODN (1 μg/mL). To measure the inhibition of TLR4 signaling, BMDMs were treated with 3 μM CLI-095 (InvivoGen) for 6 h followed by treatment with INT or LPS for 2 h. To test for contamination of reagents with LPS, BMDMs were treated with INT or TLR ligands in the presence or absence of 1 μg/mL polymyxin B (PMB) (InvivoGen).

### Mice and cell culture

C57BL/6 mice, myeloid differentiation factor 88 (MyD88)-knockout (KO) mice on a C57BL/6 background, and Toll/IL-1 receptor (TIR) domain-containing adaptor (TRIF)-KO mice (C57BL/6J-Ticam1LPS/J) were maintained at the animal facility of the National Institute of Child Health and Development (NICHD), National Institutes of Health, Bethesda, MD, USA, as described previously (10). MyD88-KO mice were provided by Dr. Shizuo Akira (8). TRIF-KO mice were purchased from The Jackson Laboratories. All experiments involving mice were performed in accordance with the guidelines of the NICHD Institutional Animal Care and Use Committee (study protocols 14-044 and 17-044).

BMDMs were prepared from C57BL/6 mice (1–3 months old) as described (23). Briefly, BM cells were cultured in Dulbecco’s modified eagle medium/F12 medium (Corning, NY, United States) supplemented with 10% fetal bovine serum, 1% (v/v) of nonessential amino acid (Gibco, CA, United States), sodium pyruvate (Gibco), l-gultamine (Gibco), 10 mM of 2-mercaptoethanol (Gibco), antibiotics (penicillin or streptomycin) (Gibco), and supernatants harvested from cultures of the murine fibroblast cell line L929 cells as a source of macrophage colony-stimulating factor (20% [v/v]). On days 5–7, the cells (2 × 10^5^/well) were added to 24-well plates and primed with 100 U/mL of purified recombinant mouse IFN-β (R&D Systems, Minneapolis, MN, United States) overnight (24).

BM-derived dendritic cells (DCs) were cultured in RPMI 1640 medium (Gibco) supplemented with 10% fetal bovine serum, 1% (v/v) of nonessential amino acids (Gibco), sodium pyruvate (Gibco), l-gultamine (Gibco), 10 mM of 2-mercaptoethanol (Gibco), and antibiotics (penicillin/streptomycin) (Gibco) with 200 ng/mL of human recombinant fms-like tyrosine kinase 3 ligand (Flt3L) (PeproTech, Rocky Hill, NJ, United States) as reported (25). This culture system routinely generated a cell population comprising approximately 15% CD11c+ (Biolegend, San Diego, CA, United States), PDCA-1+ (eBioScience, San Diego, United States), and B220+ (Biolegend) plasmacytoid DCs (pDCs). The pDCs used for stimulation were isolated using a BD FACS Aria. Murine embryonic fibroblasts (MEFs) were prepared from 15-day-old embryos harvested from wild-type C57BL/6 mice (26).

### RT-qPCR detection of IFN mRNA

Ifnα, Ifnβ, and Il1β mRNAs were measured using RT-qPCR as described (10, 23). Transcript levels were normalized to those of Gapdh, and the data are expressed as fold-induction. The primers were as follows (5′-3′):

Gapdh (Fw): GTGTTCCTACCCCCAATGT or CGTCCCGTAGACAAAATGGT

Gapdh (Rv): TGTCATCATACTTGGCAGGTTTC or TTGATGGCAACAATCTCCAC

Ifna (Fw): TCTGATGCAGCAGGTGGG

Ifna (Rv): AGGGCTCTCCAGACTTCTGCTCTG

*The primers amplified sequences encoding all isoforms IFNα isoforms (9, 10).

Ifnb (Fw): ACAGCACCAGCTTCTTCATCAG

Ifnb (Rv): TCTTCAAAGGCTTCATCTGCAA

Il1b (Fw): AGTTGACGGACCCCAAAGA

Il1b (Rv): GGACAGCCCAGGTCAAAGG

### Protein detection of IFN

IFNβ protein levels were measured using an enzyme-linked immunosorbent assay (ELISA) busing the VeriKine-HS Mouse IFN Beta Serum ELISA Kit (PBL Assay Science, Piscataway, NJ, United States) as described (10, 23).

### Encephalomyocarditis virus infection and assays of viral replication

MEFs (1 × 10^6^ per plate) were added to a 24-well plate and cultured for 18 h. Cells were treated with INT, TLR ligands, or supernatants from BMDMs treated with these agents before or after infection. The cells were washed and incubated for 40–57 h. Virus-induced cytolysis was visualized using crystal violet staining. Virus titers in supernatants of MEFs infected with encephalomyocarditis virus (EMCV) (ATCC VR-1479) were determined using a plaque assay as described (26).

## Results

### INT induces the expression of IFNβ and IFNα in BMDMs

**Fig. 1:**
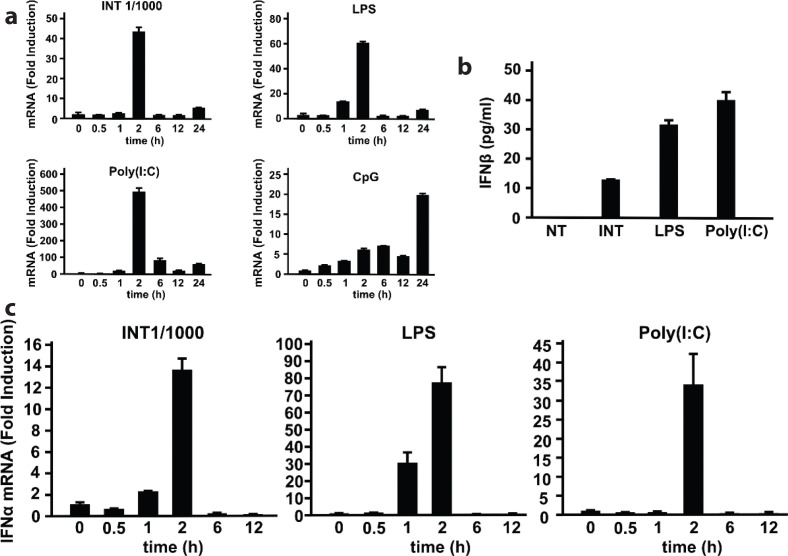
Internatural (INT) induces the expression of type I interferons (IFNs) in bone marrow-derived macrophages (BMDMs). (a) BMDMs were treated with INT (diluted 1:1,000), lipopolysaccharide (LPS) (200 ng/mL), polyinosine-polycytidylic acid [poly(I:C)] (1 μg/mL), and CpG (ODN 1826, 1 μg/mL). Ifnβ transcripts were measured using RT-qPCR and expressed as fold-induction after normalization to those of Gapdh. The values represent the average of technical duplicates ± SD. Hereafter, results are representative of three independent experiments. (b) Enzyme-linked immunosorbent assay analysis of IFNβ proteins in supernatants harvested after 24 h from cultures of BMDMs treated with INT, LPS, or poly(I:C) at the abovementioned concentrations. The values represent the average of technical duplicates ± SD. Hereafter, results are representative of two independent experiments. (c) Ifnα mRNA levels in BMDMs treated with INT and other ligands. The values represent the average of technical duplicates ± SD. Hereafter, results are representative of three independent experiments.

We first determined the ability of INT to induce the expression of Ifnβ mRNA in BMDMs. Fig. 1a presents a comparison of the temporal levels of Ifnβ in the presence of INT, LPS, poly(I:C), and CpG. INT, diluted 1:1,000, was optimal for inducing Ifnβ expression in BMDMs, in that at this dilution of INT, IFNβ mRNA levels reached >40-fold higher at 2 h. LPS and poly(I:C) also induced IFNβ peaking at 2 h. In contrast, CpG did not significantly induce IFNβ during the first 2 h incubation, but did so later at 24 h. The highest Ifnβ induction was observed with poly(I:C), followed by LPS, INT, and CpG. Fig. 1b shows the amounts of Ifnβ protein in supernatants from BMDMs treated with above agents. Although lower than the amounts produced by LPS and poly(I:C), INT-treated cells also produced IFNβ by >10 pg/mL. We next studied induction of IFNα by INT. Data in Fig. 1c show that INT induced IFNα mRNA in BMDMs, albeit at lower than that by LPS and poly(I:C).

### INT signals through TLR4

TLR4 binds LPS and signals through two adaptors, TRIF and MyD88, whereas TLR3 that recognizes poly(I:C) exclusively signals through TRIF (23, 27–30). Other TLRs, including TLR9, bind CpG and utilize MyD88, but not TRIF (27, 28). Ligand binding and adaptor activation lead to transcription of IFNβ gene, followed by the subsequent induction of multiple IFNα genes (9).

**Fig. 2:**
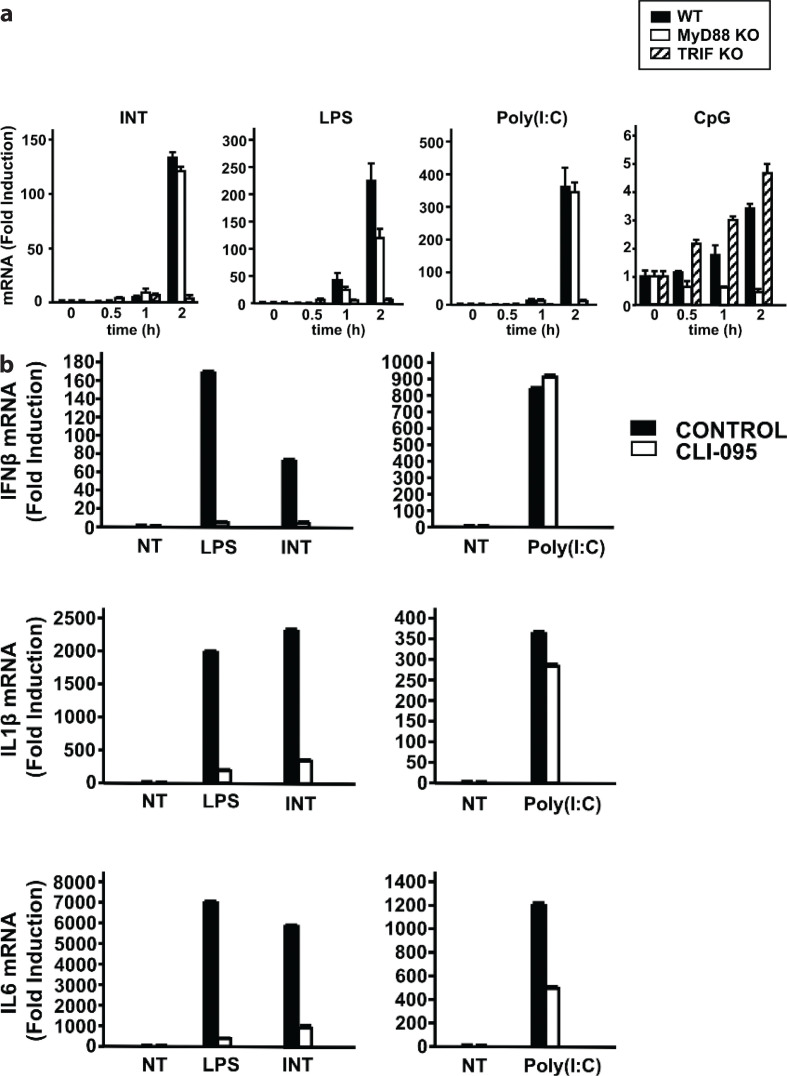
Internatural (INT) signals through TLR4. The values represent the average of technical duplicates ± SD. Hereafter, results are representative of two independent experiments. (a) Induction of Ifnβ expression by INT, lipopolysaccharide (LPS), polyinosine-polycytidylic acid [poly(I:C)], and CpG in bone marrow-derived macrophages (BMDMs) isolated from wild-type C57BL/6 mice, MyD88-KO mice, or TRIF-KO mice (C57BL/6 background). BMDMs were treated with INT (diluted 1:1,000), LPS (200 ng/mL), poly(I:C) (1 μg/mL), or CpG (1826 ODN, 1 μg/mL) for 0–2 h. Ifnβ mRNA levels were measured as described in Fig. 1. (b) The effect of the TLR4 inhibitor, CLI-095, on the induction of Ifnβ (top), Il1β (middle), and IL-6 (bottom) expressions. BMDMs were treated for 6 h with INT, LPS, or poly(I:C) in the presence or absence of CLI-095 (3 μM).

To identify the signaling pathway that mediates IFN induction by INT, we tested BMDMs from mice lacking the TLR adaptors MyD88 or TRIF. As shown in Fig. 2a (left panel), INT induced IFNβ in wild type (WT) cells by more than 100-fold at 2 h. INT induction of IFNβ was much lower in TRIF-KO cells than in WT cells. On the other hand, IFNβ induction was only slightly reduced in MyD88-KO cells. LPS also induced IFNβ mRNA in WT cells by more than 200-fold after 2 h (Fig. 2a, mid-left panel). Like INT, this induction was markedly reduced in TRIF-KO cells, but modestly in MyD88-KO cells, which is in agreement with the report by Palsson-McDermott and O’Neill who showed that LPS signals to TRIF and activates IFN by IRF3, while nuclear factor-kappaB (NF-κB) signals via MyD88 to activate proinflammatory cytokines such as TNF and IL-1 (30). Previously, we also observed similar strong dependence of IFN induction on TRIF with another TLR4 ligand (23, 29). Poly (I:C), a ligand for TLR3, induced IFNβ by more than 300-fold at 2 h in WT cells, and this induction was inhibited in TRIF-KO cells as expected. In contrast, CpG, a ligand for TLR9, did not induce IFNβ expression at significant levels in 2 h (see the difference in scale in the Y axis) (Fig 2a, right panel). The peak induction was observed later at 24 h (data not shown). Together, the time course of IFNβ induction by INT and strong dependence on the TRIF adaptor pointed to a similarity to LPS induction of IFN, suggesting that INT may signal through TLR4.

To further test the possibility that INT acts through TLR4, we examined the effects of the TLR4-specific inhibitor, CLI-095 (31, 32). CLI-095 was added to BMDMs at 6 h before adding INT or other TLR ligands. CLI-095 completely inhibited the induction of Ifnβ by INT and LPS (Fig. 2b, left panel). However, CLI-095 did not inhibit poly(I:C) induction of IFNβ mRNA (Fig. 2b, right panel). We also observed that INT induced IL-1β and IL-6 mRNA, in addition to IFNβ similar to LPS and poly(I:C). Induction of IL-1β and IL-6 by INT and LPS was also inhibited by CLI-095, but not the induction by poly(I:C). These results show that INT signals through TLR4.

**Fig. 3:**
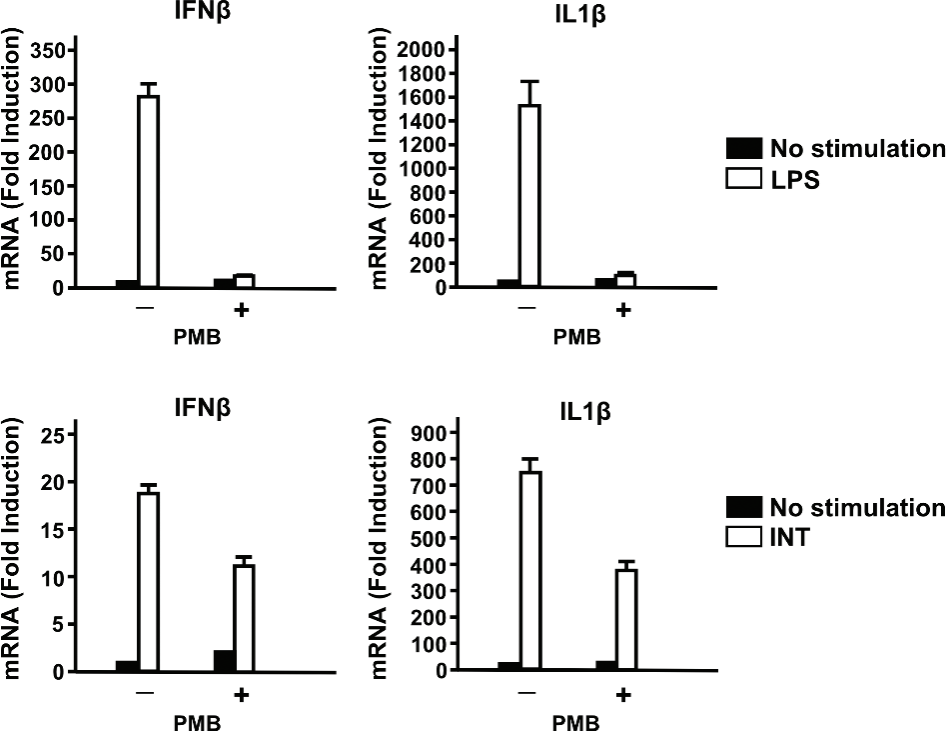
Ifnβ and Il1β are induced upon internatural (INT) stimulation, not lipopolysaccharide (LPS) contamination. The effect of polymyxin B (PMB) on the induction of Ifnβ and Il1β expressions by INT or LPS is shown. Bone marrow-derived macrophages were treated for 2 h with INT or LPS with or without PMB. The values represent the average of technical duplicates ± SD. Hereafter, results are representative of three independent experiments.

To exclude the possibility that our INT preparations contained LPS and CLI-095 inhibited the activity of contaminating LPS, we tested the effect of PMB, which neutralizes LPS activity by binding to its lipid A moiety (33, 34). PMB (1 ng/mL) abolished LPS-induced Ifnβ and IL-1β expression but had no effect on the induction of these cytokines by INT (Fig. 3). These results show that the activation of TLR4 and the induction of IFNβ and other proinflammatory cytokines by INT are not due to LPS contamination.

### INT induces IFNa and IFNb moderately in pDCs and MEFs

**Fig. 4:**
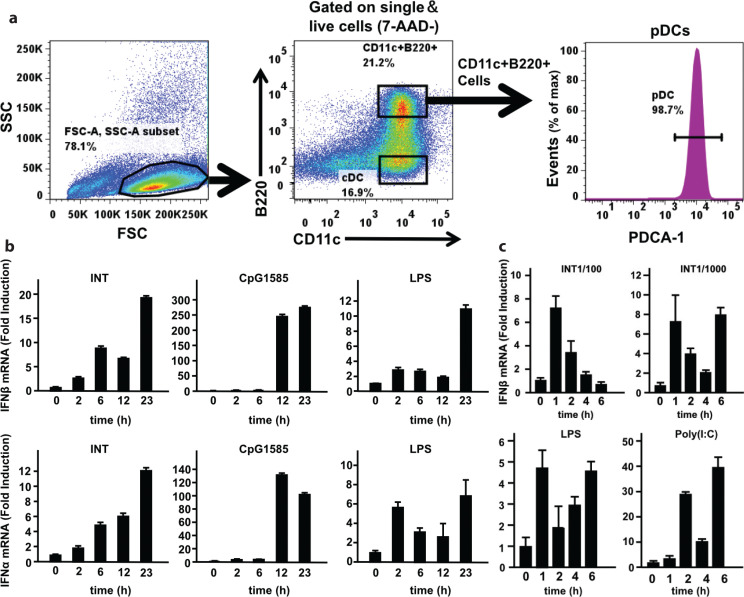
Effects of internatural (INT) on the induction of type I interferon (IFN) expression in plasmacytoid dendritic cells (pDCs) and murine embryonic fibroblasts (MEFs). (a) Fluorescence-activated cell sorting analysis of pDCs generated from bone marrow culture using Flt3L. The population of CD11c+, PDCA-1+, and B220+ cells (circled) was sorted and tested for the induction of IFN expression by INT. (b) RT-qPCR analysis of type I IFN mRNA in pDCs treated with INT and other ligands. Data represent the average of two independent experiments ± SD. (c) MEFs were treated with ligands, and Ifnβ mRNA levels were measured as described in (b). The values represent the average of technical duplicates ± SD. Hereafter, results are representative of three independent experiments.

To assess the range of cells in which INT induces IFNs, we tested plasmacytoid DCs (pDCs) and MEFs for their ability to induce type I IFNs by INT. pDCs produce high levels of type I IFNs in response to viral infection and other stimuli (35, 36) but respond to LPS moderately because they express TLR4 at lower levels than in BMDMs (25, 35). MEFs and other fibroblasts express varying levels of TLR4 and mildly respond to LPS to induce type I IFNs (37). We generated pDCs from BM cells in the presence of Flt3L and isolated using fluorescence-activated cell sorting (FACS) (38). The FACS profile in Fig. 4a shows CD11c+, PDCA-1+, and B220+ pDC populations. pDCs were treated with INT, LPS, or poly(I:C) and tested for induction of IFNβ and IFNα (Fig. 4b). INT and LPS induced modest amounts of IFNs, which peaked at 1 day later (23 h) (Fig. 4b, left and right panels). In contrast, CpG induced both IFNs at much higher levels, peaking at 12 h and 23 h (Fig. 4b, middle panel).

As shown in Fig. 4c, top panel, INT also induced moderate levels of IFNβ in MEFs at both 1/100 and 1/1,000 dilutions, which displayed different time courses. This induction patten was similar to that by LPS, although the overall levels of IFNβ were lower than those by INT (Fig. 4c, bottom panel). The weak IFN induction by LPS observed here is consistent with a previous report (37). However, poly(I:C) induced IFNβ at considerably higher levels (Fig 4c, bottom panel). These results indicate that INT stimulates type I IFN expression in multiple cell types in a manner consistent with TLR4-mediated signaling.

### High-MW components of INT activate the expression of type I IFN genes

**Fig. 5:**
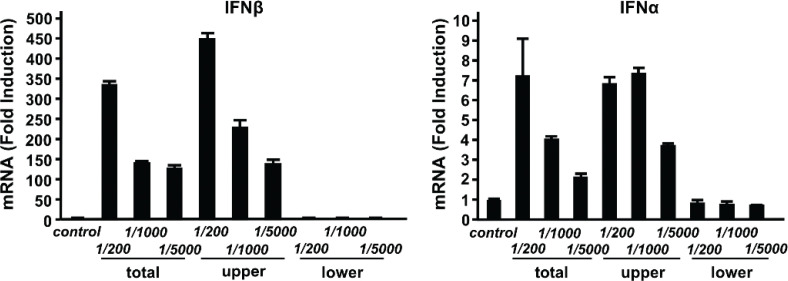
High-molecular-weight components in internatural (INT) stimulate type I interferon (IFN) induction. INT was separated into high- and low-molecular-weight components using an ultrafiltration system with a 10,000 molecular weight cut-off. Each fraction was tested for the ability to induce the expressions of Ifnβ and Ifnα mRNAs in bone marrow-derived macrophages. Total: unfractionated INT; upper: >10,000 MW; lower: <10,000 MW. The values represent the average of technical duplicates ± SD. Hereafter, results are representative of two independent experiments.

To begin assessing the molecular properties of INT, we investigated approximate MWs of its active components. We used an ultracentrifugation system to fractionate INT into components larger or smaller than 10,000 MW. The two fractions were tested for induction of IFNβ and IFNα (Fig. 5). We found that the high-MW fraction induced both Ifnα and ifnβ at a-levels comparable to or greater than unfractionated INT. In contrast, the low-MW fraction did not induce a significant amount of either IFNα or Ifnβ.

### INT confers host resistance against EMCV infection through type I IFN

**Fig. 6:**
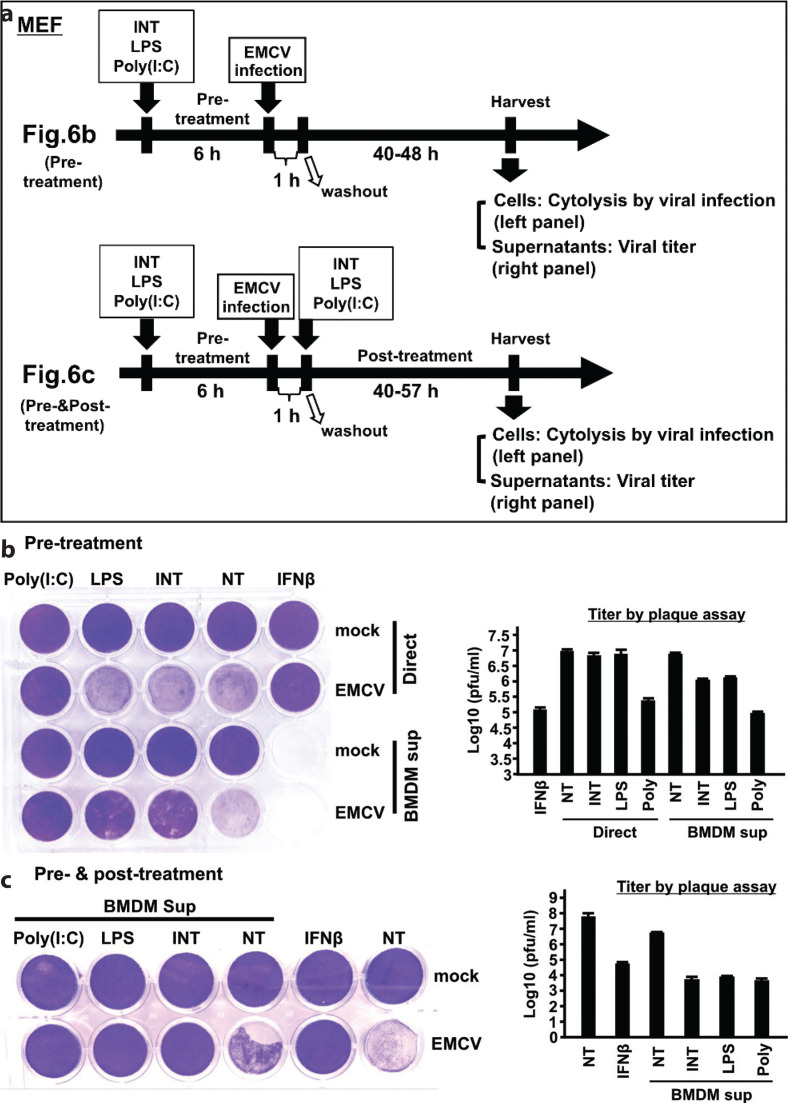
Internatural (INT) supernatants confer protection against encephalomyocarditis virus (EMCV) infection. The values represent the average of technical duplicates ± SD. Hereafter, results are representative of three independent experiments. (a) Experimental protocol. Murine embryo fibroblasts (MEFs) were treated with INT and infected with EMCV at a multiplicity of infection = 0.00001 per well for 1 h. Virus-induced cell lysis was monitored 40–57 h later using crystal violet staining, and virus titers in MEF supernatants were measured using a plaque assay. (b) Left: MEFs in 24-well plates at (1 × 10^5^ cells per well) were treated with INT (direct, upper) or bone marrow-derived macrophages (BMDMs) supernatants with the indicated ligands for 6 h. Cells were then infected with EMCV for 1 h and incubated for 40–48 h. Virus-induced cell lysis was monitored by Crystal Violet stain as above. NT: no treatment. IFNβ served as a control. Right: plaque assay of virus titers of supernatants. (c) BMDM supernatants were added to MEFs immediately after EMCV infection, and cells were cultured thereafter for 40–57 h. Cytolysis and plaque assays were performed as described in (b).

To ascertain whether INT has the property to protect cells from viral infection, we next investigated its effects on EMCV infection in MEFs (Fig. 6a) (39). Antiviral activity of INT was assessed in MEFs by two ways: first, MEFs directly exposed to INT; second, MEFs were exposed to supernatant of INT-treated BMDMs. The activity of INT was compared with that of LPS and poly(I:C) that were administered as INT (direct or supernatants). As shown in the experimental diagram in Fig. 6a, MEFs pretreated with INT, LPS, and poly(I:C), or BMDM supernatants were then infected with EMCV for 40–48 h, and antiviral activity was assessed by MEF cytolysis or measuring viral titer from supernatants of virus-infected MEFs. Results of virus-mediated cytolysis are presented in Fig. 6b. Viral titers recovered from each experiment are shown in Fig. 6c. As a positive control, we tested IFNβ, and PBS as a negative control (NT in Fig. 6b and c). Direct addition of INT or LPS led to only slight protection against viral infection as evidenced by measurable cytolysis. Viral titers were only slightly lower than NT control. As expected, the addition of IFNβ led to complete protection against cytolysis and several magnitude lower viral titers. Direct addition of poly(I:C), on the other hand, gave higher antiviral activity compared to INT and LPS, as it led lower cytolysis and lower viral titer, the results in line with its greater ability to induce IFNβ than INT or LPS (Fig. 4c). Although INT displayed a modest antiviral activity when added directly, supernatants from INT-treated BMDMs gave a respectable protection (Fig. 6b), in that cytolysis was much reduced and viral titers were approximately 10-fold lower than that in NT, untreated control. To corroborate INT’s antiviral activity, we also tested BMDM supernatants by adding INT both prior to and after EMCV infection (Fig. 6c). We found that continuous presence of INT in BMDMs led to be even greater antiviral activity, as cytolysis and viral titers were further reduced, which were approximately 1,000-fold lower than that in NT. Thus, INT, by producing type I IFNs directly protect virus-infected MEFs, when added to BMDMs indirectly, protect a third party MEFs by secreting IFN in the supernatants.

## Discussion

We report here that the herbal extracts INT, widely used by the public in Japan and elsewhere to enhance innate immunity, induce the expression of type I IFNs in BMDMs and other cell types. Through this activity, INT protected MEFs from viral infection. Consistent with these results, Kojima and his associates observed more than 40 years ago that INT inhibits vaccinia virus growth in a rabbit model (20, 21). Type I IFNs exert broad inhibitory activity against infection by viruses, bacteria, and parasites (2). Type I IFNs enhance innate and adaptive immunity and modulate the pathogenesis of chronic diseases, including inflammation, cardiovascular diseases, autoimmune diseases, and even cancer (11, 12, 40).

INT did not contain LPS (endotoxin) and its IFN-inducing activity resided in a high-MW fraction of the extracts. INT exerted its effects through the conserved TLR pathway, specifically through TLR4. This conclusion was attained by the abrogation of IFN induction by the TLR4-specific inhibitor, CLI-095.

LPS is the most widely studied TLR4 ligand (5–7). LPS, a component of Gram-negative bacteria, is widely present in the environment and can be generated internally by infection, which can cause serious health threats (41, 42). Depending on the amount, it causes acute endotoxin shock and sepsis, a life-threatening condition. Furthermore, a single LPS exposure can lead to so-called ‘tolerance’, where the host undergoes a profound unresponsive state toward the subsequent LPS exposure, which makes the host vulnerable to infection. Lipid A is the active moiety of LPS, which binds to TLR4 in a complex with MD-2 that dimerizes after engagement of lipid A (43). In addition to IFNs, LPS induces proinflammatory cytokines, such as IL-1 and IL-6, the source of inflammation. In this study, INT and LPS displayed similar strong dependence on TRIF, but it marginally required on MyD88 for IFN induction. INT and LPS displayed similar time course of IFN induction and similar cell-type specificities. These results indicated that INT signals through TLR4, which was further substantiated by the identical sensitivity to TLR4-specific inhibitor, CLI-095. It is, thus, likely that INT binds to TLR4/MD-2 and activates IRF3/IRF7 and NF-κB to induce IFNs and inflammatory cytokines (5–7). However, the possibility that INT acts indirectly on TLR4 to influence downstream signaling cannot be excluded (44).

A broad array of natural ligands including plant derivatives and synthetic compounds interacts with TLR4/MD-2 (43, 45), such as green tea, curcumin, licorice, and synthetic neoseptin-1. These ligands act as agonists or antagonists for TLR4 (43). It may be possible that TLR4 is frequently engaged by naturally occurring herbal ligands that modulate transcriptional outcomes. Further analysis of the biological activities of INT and other ligands may provide a deeper and broader understanding of innate immune responses.

## Conflict of interest and funding

All authors declare no conflict of interest. This work was supported by the intermural program of NICHD, NIH, ZIA HD001310-34.

## References

[1] Kaur S, Uddin S, Platanias LC. The PI3’ kinase pathway in interferon signaling. J Interferon Cytokine Res 2005; 25: 780–7. doi: 10.1089/jir.2005.25.780 16375606

[2] MacMicking JD. Interferon-inducible effector mechanisms in cell-autonomous immunity. Nat Rev Immunol 2012; 12: 367–82. doi: 10.1038/nri3210 22531325 PMC4150610

[3] Chen J, Baig E, Fish EN. Diversity and relatedness among the type I interferons. J Interferon Cytokine Res 2004; 24: 687–98. doi: 10.1089/jir.2004.24.687 15684736

[4] van Pesch V, Lanaya H, Renauld JC, Michiels T. Characterization of the murine alpha interferon gene family. J Virol 2004; 78: 8219–28. doi: 10.1128/jvi.78.15.8219-8228.2004 15254193 PMC446145

[5] Kawai T, Akira S. Toll-like receptor and RIG-I-like receptor signaling. Ann N Y Acad Sci 2008; 1143: 1–20. doi: 10.1196/annals.1443.020 19076341

[6] O’Neill LA, Bowie AG. The family of five: TIR-domain-containing adaptors in Toll-like receptor signalling. Nat Rev Immunol 2007; 7: 353–64. doi: 10.1038/nri2079 17457343

[7] O’Neill LA, Golenbock D, Bowie AG. The history of Toll-like receptors – redefining innate immunity. Nat Rev Immunol 2013; 13: 453–60. doi: 10.1038/nri3446 23681101

[8] Adachi O, Kawai T, Takeda K, Matsumoto M, Tsutsui H, Sakagami M, et al. Targeted disruption of the MyD88 gene results in loss of IL-1- and IL-18-mediated function. Immunity 1998; 9: 143–50. doi: 10.1016/s1074-7613(00)80596-8 9697844

[9] Marié I, Durbin JE, Levy DE. Differential viral induction of distinct interferon-alpha genes by positive feedback through interferon regulatory factor-7. Embo J 1998; 17: 6660–9. doi: 10.1093/emboj/17.22.6660 9822609 PMC1171011

[10] Tailor P, Tamura T, Kong HJ, Kubota T, Kubota M, Borghi P, et al. The feedback phase of type I interferon induction in dendritic cells requires interferon regulatory factor 8. Immunity 2007; 27: 228–39. doi: 10.1016/j.immuni.2007.06.009 17702615 PMC2768351

[11] Corrales L, Glickman LH, McWhirter SM, Kanne DB, Sivick KE, Katibah GE, et al. Direct activation of STING in the tumor microenvironment leads to potent and systemic tumor regression and immunity. Cell Rep 2015; 11: 1018–30. doi: 10.1016/j.celrep.2015.04.031 25959818 PMC4440852

[12] Doherty MR, Cheon H, Junk DJ, Vinayak S, Varadan V, Telli ML, et al. Interferon-beta represses cancer stem cell properties in triple-negative breast cancer. Proc Natl Acad Sci USA 2017; 114: 13792–7. doi: 10.1073/pnas.1713728114 29229854 PMC5748193

[13] Ji HF, Li XJ, Zhang HY. Natural products and drug discovery. Can thousands of years of ancient medical knowledge lead us to new and powerful drug combinations in the fight against cancer and dementia? EMBO Rep 2009; 10: 194–200. doi: 10.1038/embor.2009.12 19229284 PMC2658564

[14] Petrovska BB. Historical review of medicinal plants’ usage. Pharmacogn Rev 2012; 6: 1–5. doi: 10.4103/0973-7847.95849 22654398 PMC3358962

[15] Lin CW, Wu CF, Hsiao NW, Chang CY, Li SW, Wan L, et al. Aloe-emodin is an interferon-inducing agent with antiviral activity against Japanese encephalitis virus and enterovirus 71. Int J Antimicrob Agents 2008; 32: 355–9. doi: 10.1016/j.ijantimicag.2008.04.018 18701259 PMC7126984

[16] Yue Y, Li Z, Li P, Song N, Li B, Lin W, et al. Antiviral activity of a polysaccharide from *Laminaria japonica* against enterovirus 71. Biomed Pharmacother 2017; 96: 256–62. doi: 10.1016/j.biopha.2017.09.117 28987950

[17] Lin LT, Hsu WC, Lin CC. Antiviral natural products and herbal medicines. J Tradit Complement Med 2014; 4: 24–35. doi: 10.4103/2225-4110.124335 24872930 PMC4032839

[18] Huang XY, Huang ZL, Wang L, Xu YH, Huang XY, Ai KX, et al. Herbal compound ‘Songyou Yin’ reinforced the ability of interferon-alfa to inhibit the enhanced metastatic potential induced by palliative resection of hepatocellular carcinoma in nude mice. BMC Cancer 2010; 10: 580. doi: 10.1186/1471-2407-10-580 20969807 PMC2976755

[19] Han C, Kawata M, Hamada Y, Kondo T, Wada J, Asano K, et al. Analyses of the possible anti-tumor effect of yokukansan. J Nat Med 2019; 73: 468–79. doi: 10.1007/s11418-019-01283-x 30739283

[20] Kojima Y, Hashimoto H, Shibukawa N. Priming effect of interferon on the production of endotoxin-induced interferon in rabbits and rabbit lymphoid cells. Ann N Y Acad Sci 1980; 350: 632. doi: 10.1111/j.1749-6632.1980.tb20693.x

[21] Hashimoto H, Shibukawa N, Kojima Y. The mode of production of endotoxin-induced interferon in rabbit lymphoid cell cultures. II. Priming effect of interferon on apparently noninducible cells. Microbiol Immunol 1979; 23: 1033–6. doi: 10.1111/j.1348-0421.1979.tb00533.x 514095

[22] Ozato K, Uno K, Iwakura Y. Another road to interferon: Yasuichi Nagano’s journey. J Interferon Cytokine Res 2007; 27: 349–52. doi: 10.1089/jir.2007.9988 17523866

[23] Ayithan N, Bradfute SB, Anthony SM, Stuthman KS, Dye JM, Bavari S, et al. Ebola virus-like particles stimulate type I interferons and proinflammatory cytokine expression through the toll-like receptor and interferon signaling pathways. J Interferon Cytokine Res 2014; 34: 79–89. doi: 10.1089/jir.2013.0035 24102579 PMC3924795

[24] Ivashkiv LB, Donlin LT. Regulation of type I interferon responses. Nat Rev Immunol 2014; 14: 36–49. doi: 10.1038/nri3581 24362405 PMC4084561

[25] Tsujimura H, Tamura T, Ozato K. Cutting edge: IFN consensus sequence binding protein/IFN regulatory factor 8 drives the development of type I IFN-producing plasmacytoid dendritic cells. J Immunol 2003; 170: 1131–5. doi: 10.4049/jimmunol.170.3.1131 12538667

[26] Kamada R, Yang W, Zhang Y, Patel MC, Yang Y, Ouda R, et al. Interferon stimulation creates chromatin marks and establishes transcriptional memory. Proc Natl Acad Sci USA 2018; 115: E9162–71. doi: 10.1073/pnas.1720930115 30201712 PMC6166839

[27] Yamamoto M, Sato S, Hemmi H, Uematsu S, Hoshino K, Kaisho T, et al. TRAM is specifically involved in the Toll-like receptor 4-mediated MyD88-independent signaling pathway. Nat Immunol 2003; 4: 1144–50. doi: 10.1038/ni986 14556004

[28] Yamamoto M, Sato S, Hemmi H, Hoshino K, Kaisho T, Sanjo H, et al. Role of adaptor TRIF in the MyD88-independent toll-like receptor signaling pathway. Science 2003; 301: 640–3. doi: 10.1126/science.1087262 12855817

[29] Takeuchi O, Akira S. Pattern recognition receptors and inflammation. Cell 2010; 140: 805–20. doi: 10.1016/j.cell.2010.01.022 20303872

[30] Pålsson-McDermott EM, O’Neill LA. Signal transduction by the lipopolysaccharide receptor, Toll-like receptor-4. Immunology 2004; 113: 153–62. doi: 10.1111/j.1365-2567.2004.01976.x 15379975 PMC1782563

[31] Ii M, Matsunaga N, Hazeki K, Nakamura K, Takashima K, Seya T, et al. A novel cyclohexene derivative, ethyl (6R)-6-[N-(2-Chloro-4-fluorophenyl)sulfamoyl]cyclohex-1-ene-1-carboxylate (TAK-242), selectively inhibits toll-like receptor 4-mediated cytokine production through suppression of intracellular signaling. Mol Pharmacol 2006; 69: 1288–95. doi: 10.1124/mol.105.019695 16373689

[32] Kawamoto T, Ii M, Kitazaki T, Iizawa Y, Kimura H. TAK-242 selectively suppresses Toll-like receptor 4-signaling mediated by the intracellular domain. Eur J Pharmacol 2008; 584: 40–8. doi: 10.1016/j.ejphar.2008.01.026 18299127

[33] Domingues MM, Inácio RG, Raimundo JM, Martins M, Castanho MA, Santos NC. Biophysical characterization of polymyxin B interaction with LPS aggregates and membrane model systems. Biopolymers 2012; 98: 338–44. doi: 10.1002/bip.22095 23193598

[34] Cardoso LS, Araujo MI, Góes AM, Pacífico LG, Oliveira RR, Oliveira SC. Polymyxin B as inhibitor of LPS contamination of Schistosoma mansoni recombinant proteins in human cytokine analysis. Microb Cell Fact 2007; 6: 1. doi: 10.1186/1475-2859-6-1 17201926 PMC1766364

[35] Liu YJ. IPC: professional type 1 interferon-producing cells and plasmacytoid dendritic cell precursors. Annu Rev Immunol 2005; 23: 275–306. doi: 10.1146/annurev.immunol.23.021704.115633 15771572

[36] Dalod M, Salazar-Mather TP, Malmgaard L, Lewis C, Asselin-Paturel C, Brière F, et al. Interferon alpha/beta and interleukin 12 responses to viral infections: pathways regulating dendritic cell cytokine expression in vivo. J Exp Med 2002; 195: 517–28. doi: 10.1084/jem.20011672 11854364 PMC2193614

[37] Yoshimi R, Chang TH, Wang H, Atsumi T, Morse HC, 3rd, Ozato K. Gene disruption study reveals a nonredundant role for TRIM21/Ro52 in NF-kappaB-dependent cytokine expression in fibroblasts. J Immunol 2009; 182: 7527–38. doi: 10.4049/jimmunol.0804121 19494276 PMC2803686

[38] Brawand P, Fitzpatrick DR, Greenfield BW, Brasel K, Maliszewski CR, De Smedt T. Murine plasmacytoid pre-dendritic cells generated from Flt3 ligand-supplemented bone marrow cultures are immature APCs. J Immunol 2002; 169: 6711–9. doi: 10.4049/jimmunol.169.12.6711 12471102

[39] Carocci M, Bakkali-Kassimi L. The encephalomyocarditis virus. Virulence 2012; 3: 351–67. doi: 10.4161/viru.20573 22722247 PMC3478238

[40] Crow MK. Type I interferon in organ-targeted autoimmune and inflammatory diseases. Arthritis Res Ther 2010; 12 Suppl 1(Suppl 1): S5. doi: 10.1186/ar2886 21303493 PMC2991778

[41] Munford RS. Sensing gram-negative bacterial lipopolysaccharides: a human disease determinant? Infect Immun 2008; 76: 454–65. doi: 10.1128/iai.00939-07 18086818 PMC2223455

[42] Biswas SK, Lopez-Collazo E. Endotoxin tolerance: new mechanisms, molecules and clinical significance. Trends Immunol 2009; 30: 475–87. doi: 10.1016/j.it.2009.07.009 19781994

[43] Peri F, Calabrese V. Toll-like receptor 4 (TLR4) modulation by synthetic and natural compounds: an update. J Med Chem 2014; 57: 3612–22. doi: 10.1021/jm401006s 24188011 PMC4040398

[44] Lancaster GI, Langley KG, Berglund NA, Kammoun HL, Reibe S, Estevez E, et al. Evidence that TLR4 is not a receptor for saturated fatty acids but mediates lipid-induced inflammation by reprogramming macrophage metabolism. Cell Metab 2018; 27: 1096–110.e5. doi: 10.1016/j.cmet.2018.03.014 29681442

[45] Wang Y, Su L, Morin MD, Jones BT, Whitby LR, Surakattula MM, et al. TLR4/MD-2 activation by a synthetic agonist with no similarity to LPS. Proc Natl Acad Sci USA 2016; 113: E884–93. doi: 10.1073/pnas.1525639113 26831104 PMC4763747

